# Macrophage activation syndrome during Crohn´s disease: a case report

**DOI:** 10.11604/pamj.2022.42.97.29361

**Published:** 2022-06-06

**Authors:** Oussama Kharmach, Nawal Lagdali, Imane Benelbarhdadi, Mohammed Borahma, Fatima-Zohra Ajana

**Affiliations:** 1 Department C of Hepato-gastroenterology, Ibn Sina University Hospital, Mohammed V University, Rabat, Morocco

**Keywords:** Macrophage activation syndrome, Crohn’s disease, infection, hemophagocytosis, case report

## Abstract

The macrophage activation syndrome (MAS) is a rare but potentially fatal disease. We report two cases of Crohn´s disease, one under 5-aminosalicylic acid and the other under corticosteroid, having developed MAS. The first was a male patient hospitalized for high fever, clinical examination found fever and left inguinal lymph node. The second case was a female patient hospitalized for asthenia, clinical examination found fever and oral mycosis. The biology tests revealed bi/pancytopenia, hyperferritinemia, hypofibrinogenemia. The myelogram showed hemophagocytosis. The identified infectious cause was Klebsiella pneumoniae in the urinary tract in one patient and oral Candida albicans in the other one. Thoraco-abdomino-pelvic computed tomography (CT) scan showed a thickening of the bladder in the male patient, and eliminated a deep infection and tumour cause in the female patient. The diagnosis of MAS was made, and both patients were placed on broad-spectrum antibiotic, in addition to local antifungal treatment and corticosteroid for the female patient, but the evolution was fatal for both. In conclusion, the management of MAS should be fast and multidisciplinary, based on treatment of the causal infectious agent, immunomodulatory treatment of haemophagocytosis, symptomatic treatment and replacement of organ failure.

## Introduction

The macrophage activation syndrome (MAS) is a rare but potentially fatal disease. The diagnosis is based on a combination of non-specific clinical and biological signs, requiring cytological or histological investigation of haemophagocytosis and exhaustive etiological investigation [1]. The prognosis is unfavorable in 49% of cases, clearly showing the seriousness of this pathology. It depends on several parameters: early diagnosis, positive etiological assessment, early initiation of appropriate anti-infective therapy, associated neoplastic etiology, previous immune status (HIV, immunosuppression) [2]. The aim of our article is to draw the attention of doctors, especially gastroenterologists, to the seriousness of this syndrome, especially in immunocompromised patients, in front of evocative clinical and biological signs, and to highlight the importance of early diagnosis which is the key to its management.

## Patient and observation

### First observation

**Patient history**: a 64-year-old male patient, tobacco and alcohol weaned, with family history of Crohn´s disease in the sister. He was followed for a minor caecal non-stricturing non-penetrating Crohn´s disease under 5-aminosalicylic acid. He presented a perianal abscess and fistula which was drained with seton placement. After a month he developed a fever for nine days, hence his hospitalization. The infectious anamnesis found a notion of urinary burn.

**Clinical examination**: the patient´s general state was altered (WHO performance status grade 3), fever at 40°C, Glasgow coma scale at 15, Body Mass Index (BMI)= 20 kg/m^2^ tachycardia at 120 beats/min, tachypnea at 25 breaths/min, blood pressure at 09/06 cmHg, mobile and painless left inguinal lymph nodes of 2 cm, painless soft abdomen, clean drained anal fistula no hepato-splenomegaly.

**Figure 1:**
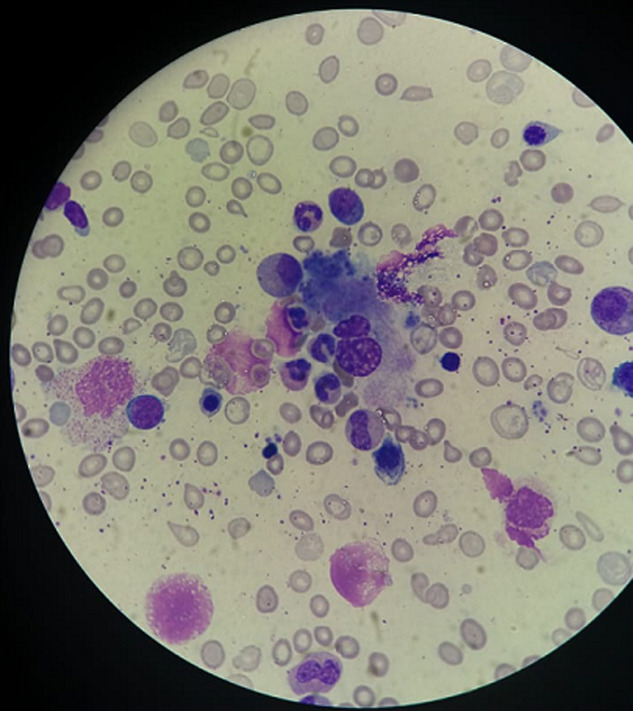
1000 x magnification image of bone marrow smear with MGG staining showing hemophagocytosis

**Diagnostic approach**: haemogram test showed anemia at 10 g/dL (MCV= 77 μm^3^, MCHC =35%), leukopenia at 520/mm^3^ (neutropenia at 0/mm^3^, lymphopenia at 510/mm^3^), normal platelet counts (170 000/mm^3^). Biochemical tests found Hyperferritinemia at 2144 ng/mL, Hypofibrinogenemia at 1g/L, elevated C-Reactive Protein (CRP) at 271 mg/L, Cytolysis (AST= 405 UI/L, ALT= 222 UI/L), cholestasis (ALP= 120 UI/L, GGT= 105 UI/L). Ionogram detected an hyponatremia at 124 mEq/L and normal potassium levels (3.6 mEq/L), normal triglyceridemia (0.9 g/L). We performed a myelogram in front of the disturbance of the red and white bloodlines that found macrophages with haemophagocytosis images (Figure 1). Infectious disease assessment in search of a causative agent revealed Klebsiella pneumonia in cytobacteriologic examination of the urine (CBEU). Other investigation tests, such as blood cultures, coproculture, viral serology A, B, C, HIV and *Treponema pallidum* haemagglutination/ venereal disease research laboratory (TPHA/VDRL) test, Hepatitis C virus Polymerase Chain Reaction (HCV PCR), Interferon Gamma Release Assays were negative. Patient was immunized by previous contact with Cytomegalovirus (CMV) and Epstein–Barr virus (EBV). Thoraco-abdomino-pelvic CT scan revealed a thickening of the bladder wall, left inguinal adenopathy of 21x15mm, minimal peritoneal effusion in the pouch of Douglas, absence of any infectious or tumour focus in the pulmonary stage.

**Diagnostic decision**: diagnosis of MAS was made in front of fever, bicytopenia, hyperferritinemia, hypofibrinogenemia and hemophagocytosis in myelogram, secondary to a urinary tract infection by Klebsiella pneumonia. The presence of five criteria of MAS, absence of hereditary family history, elimination of hematologic malignancy on myelogram with evidence of haemophagocytosis ruled out any other differential diagnosis.

**Therapeutic intervention**: the patient was transferred to the intensive care unit, where he had a nasogastric tube inserted; and the placement of a urinary and central venous catheters, and put under intravenous antibiotic therapy Ertapenem 1g/d and Piperacillin 4g-Tazobactam 0,5g/8h, Paracetamol 1g/8h, with correction of hydro-electrolytic disorders by intravenous infusion of 500cc of glucose serum 5% containing 4g of NaCl and 1g of KCl/3h.

**Evolution**: the patient died of cardiac arrest the next day.

**Informed consent**: the patient died without giving the consent.

### Second observation

**Patient history**: fourty-year-old woman, no personnel or family history. She was followed for colonic non-stricturing non-penetrating Crohn´s disease in exacerbation on oral corticosteroid 60mg/d, taken once in the morning, since four weeks. She was hospitalized for intense asthenia for a week.

**Clinical examination**: the patient had fever at 38.7°C, WHO performance status grade 3, Glasgow coma scale at 15, BMI= 22 kg/m, hemodynamically and respiratory stable, oral mycosis, no hepato-splenomegaly, painless soft abdomen.

**Diagnostic approach**: haemogram test showed anemia at 8.7g/dL (MCV= 75 µm^3^, MCHC= 29%), leucopenia at 1240/mm^2^ (neutropenia at 435/mm^3^, lymphopenia at 780/mm^3^), thrombocytopenia at 135 000/mm^3^. Biochemical tests revealed hyperferritinemia at 1730, hypofibrinogenemia= 1.1 g/L, normal triglyceridemia at 0.6 g/L, elevated C - reactive protein (CRP) at 144. Myelogram found haemophagocytosis. Infectious disease assessment was carried out. Oral sample found candida albicans. Blood cultures, coproculture, CBEU, Interferon Gamma Release Assays, Serology HCV and HIV were negative. The patient was immunised by previous contact with CMV and EBV, and by vaccination with HBV. In thoraco-abdomino-pelvic CT scan there was no infectious focus, no deep abscess or tumor.

**Diagnostic decision**: diagnosis of MAS was made in front of fever, pancytopenia, hypofibrinogenemia, hyperferritinemia, hemophagocytosis in myelogram, secondary to fungal infection by candida albicans. We have excluded any other diagnosis in the presence of criteria of MAS, absence of hereditary family history, elimination of hematologic malignancy on myelogram with evidence of haemophagocytosis.

**Therapeutic intervention**: the patient was transferred to the intensive care unit, where she had a nasogastric tube inserted; and the placement of a urinary and central venous catheter. She had methylprednisolone 60mg/d, local miconazole four times a day, intravenous antibiotic therapy Ertapenem 1g/d and Piperacillin 4g-Tazobactam 0,5g/8h, Paracetamol 1g/8h, and intravenous infusion of 500cc of glucose serum 5% containing 2g of NaCl and 1g of KCl /12h alternately with 500cc of Sodium Chloride 0.9%/12h.

**Evolution**: the evolution was good until she died on the 5^th^ day in intensive care by a septic shock.

**Informed consent**: the patient died without giving the consent.

## Discussion

The pacrophage activation syndrome is a condition first described in the 1950s but individualized more recently, mainly since Risdall´s description of post-viral haemophagocytosis in 1979 [3]. Its overall incidence in Japan has been estimated at 51.7 cases per year [4]. There are two types of haemophagocytosis [5]: primary MAS such as familial lymphohistiocytosis, Chediak-Higashi syndrome… And secondary MAS including neoplasia like lymphoma, autoimmune diseases as for example lupus, and infectious diseases for instance HIV, leptospirosis, toxoplasmosis…

Its pathophysiology involves normal but ineffective activation of the CD8/NK T cell system, leaving the causative agent and macrophages (responsible of the activation and proliferation of the same CD8 and NK T cells) to persist. The cytotoxic cells in turn stimulate macrophage activation and the loop self-amplifies in an uncontrolled manner. Various mediators (including interferon gamma, tumor necrosis factor (TNF) alpha, interleukins and macrophage colony stimulating factor) are involved in the clinical and biological manifestations of MAS [1]. The diagnosis of MAS is based on a combination of at least five of the following criteria [6]: fever, splenomegaly, bi or pancytopenia, ferritinemia ≥ 500 g/L, hypertriglyceridemia >3mmol/L, hypofibrinogenemia <1.5g/L, hemophagocytosis in bone marrow; spleen or lymph nodes, absence of neoplasia, low or no Natural Killer cell activity, soluble IL receptor-2 ≥ 2400UI/ml. The management of MAS is based on the treatment of the causal infectious agent (which is most often viral), specific immuno-modulating treatment of haemophagocytosis (corticoids associated with etoposide), symptomatic treatment and replacement of organ failure [1].

A case of macrophage activation syndrome after treatment with infliximab for penetrating Crohn´s disease was reported by Eric Chauveau *et al*. secondary to a cytomegalovirus infection, suggesting the role of infliximab in leading to an immunosuppressive condition. The evolution was favorable under corticotherapy and broad-spectrum antibiotic therapy [7]. The prognosis of MAS is, despite treatment, often still very poor: 49% of deaths in the review by Karras and Hermine [2], 28.8% in Veerakul *et al*. [8]. The mortality risk factors are [9]: age over 30, presence of disseminated intravascular coagulation, anemia with hemoglobin less than 10 g/dL and/or thrombocytopenia with a platelet count below 100 000/mm^3^, ferritin above 500 g/L, jaundice, hyperbilirubinemia, increase in alkaline phosphatase despite rapid diagnostic and therapeutic management of MAS, the prognosis of our patients was poor. The incriminating risk factors in our patients were age, anaemia and very high ferritin levels, which may have worsen the cardiac arrest and septic shock that were non-recoverable.

## Conclusion

The etiologies of MAS are numerous, dominated by viral infections. The severity of the prognosis of MAS requires an aggressive diagnostic approach and a multidisciplinary therapeutic management, collaboration of emergency physicians, haematologists and infectiologists, in order to determine the best options according to the etiology found and the severity of the condition.
